# Three-dimensional culture systems for the expansion of pluripotent embryonic stem cells

**DOI:** 10.1002/bit.22850

**Published:** 2010-11-01

**Authors:** Michael P Storm, Craig B Orchard, Heather K Bone, Julian B Chaudhuri, Melanie J Welham

**Affiliations:** 1 Centre for Regenerative Medicine, Departments of Pharmacy & Pharmacology, University of BathBath BA2 7AY, UK; 2 Chemical Engineering, University of BathBath, UK

**Keywords:** embryonic stem cells, 3D agitated suspension culture, microcarriers, pluripotency, bioreactors

## Abstract

Mouse embryonic stem cell (ESC) lines, and more recently human ESC lines, have become valuable tools for studying early mammalian development. Increasing interest in ESCs and their differentiated progeny in drug discovery and as potential therapeutic agents has highlighted the fact that current two-dimensional (2D) static culturing techniques are inadequate for large-scale production. The culture of mammalian cells in three-dimensional (3D) agitated systems has been shown to overcome many of the restrictions of 2D and is therefore likely to be effective for ESC proliferation. Using murine ESCs as our initial model, we investigated the effectiveness of different 3D culture environments for the expansion of pluripotent ESCs. Solohill Collagen, Solohill FACT, and Cultispher-S microcarriers were employed and used in conjunction with stirred bioreactors. Initial seeding parameters, including cell number and agitation conditions, were found to be critical in promoting attachment to microcarriers and minimizing the size of aggregates formed. While all microcarriers supported the growth of undifferentiated mESCs, Cultispher-S out-performed the Solohill microcarriers. When cultured for successive passages on Cultispher-S microcarriers, mESCs maintained their pluripotency, demonstrated by self-renewal, expression of pluripotency markers and the ability to undergo multi-lineage differentiation. When these optimized conditions were applied to unweaned human ESCs, Cultispher-S microcarriers supported the growth of hESCs that retained expression of pluripotency markers including SSEA4, Tra-1–60, NANOG, and OCT-4. Our study highlights the importance of optimization of initial seeding parameters and provides proof-of-concept data demonstrating the utility of microcarriers and bioreactors for the expansion of hESCs. Biotechnol. Bioeng. 2010;107:683–695. © 2010 Wiley Periodicals, Inc.

## Introduction

Embryonic stem cells (ESCs) possess two unique properties (i) they can self-renew to produce two identical undifferentiated daughter cells and (ii) they are pluripotent, meaning they have the ability to differentiate into all cell types that comprise the adult organism (Smith, 2001). This latter property of pluripotency provides the means by which cells of specific lineages can be generated and potentially used either for cell-based therapies in regenerative medicine, as a source of cells for use in the process of drug discovery or as a model for mammalian development. However, before ESCs and their derivatives can fulfil their potential the issue of culture scalability to generate clinically relevant numbers of cells has to be addressed (Thomson, 2007).

Murine ESCs (mESCs) require the presence of Leukemia inhibitory factor (LIF) to maintain their pluripotency (Smith, 2001; Smith et al., 1988; Ying et al., 2003), while with human ESCs (hESCs) there is considerable variability in the conditions required, although culture on mouse embryonic fibroblast (MEF) feeders and FGF-2 is sufficient for many lines (Vallier et al., 2005). Most ESC cultures are static two-dimensional (2D) systems, which while suitable for laboratory-scale studies, are limited by surface area availability and cumbersome for scale-up even though robotics have been successfully applied to 2D culture of hESCs (Thomas et al., 2009). Thus, introduction of another dimension is an intrinsic requirement of large-scale production rather than a by-product of the “scaling-up” process.

Some progress has been made toward the development of suitable 3D systems for the expansion of undifferentiated mESCs (Thomson, 2007). One approach has been to culture mESCs as aggregates, using shear stress to limit their size and prevent agglomeration. While 3D aggregation of mESCs is more commonly associated with induction of differentiation via embryoid bodies (Dang et al., 2004; Keller, 2005), mESCs cultured as aggregates in stirred suspension, with intermittent dispersal, retained expression of markers of pluripotency (Cormier et al., 2006; Fok and Zandstra, 2005; zur Nieden et al., 2007). An alternative strategy is to use microcarriers (van Wezel, 1967), which have the advantage of increasing the surface area to volume ratio, improving efficiency. Fok and Zandstra (2005) successfully expanded mESCs on glass-coated styrene or Cytodex-3 microcarriers, although bead-bridging led to sub-optimal culture conditions. Subsequent studies have confirmed the suitability of Cytodex-3 (Abranches et al., 2007; Fernandes et al., 2007), although Cultispher-S microcarriers also performed well (Fernandes et al., 2007). However, the issue of aggregation was not addressed in these latter studies. Another approach has been to use uncoated fibrous polyethylene terephthalate 3D matrices and conditioned media for mESC expansion (Ouyang et al., 2007).

A limited number of reports have examined the suitability of microcarriers for expansion of undifferentiated hESCs including Hillex II (Phillips et al., 2008), Matrigel-coated cellulose (Oh et al., 2009), and Cytodex-3 (Fernandes et al., 2009; Nie et al., 2009). However, information on the combination of hESCs, microcarriers and agitated suspension bioreactor systems is still extremely limited (Fernandes et al., 2009; Oh et al., 2009). The main goal of our studies was to develop a robust, well-optimized and scalable-agitated suspension culture system suitable for the expansion of pluripotent ESCs. mESCs were used as our initial model, allowing for extensive optimization of seeding and culture conditions. When optimized conditions were applied to hESCs, expansion of hESCs expressing pluripotent cell markers was achieved, providing important proof-of-principle data.

## Materials and Methods

### ESC Cell Culture

Culture and EB-based differentiation of the E14tg2a mESC line (Smith and Hooper, 1987) were carried out as described previously (Bone and Welham, 2007; Paling and Welham, 2005; Paling et al., 2004). SHEF-3 hESCs (obtained from the UK Stem Cell Bank, Potters Bar, UK) were routinely cultured on a feeder layer of mitotically inactivated MEFs in KO-DMEM supplemented with 20% (v/v) KO-SR and 4 ng/mL FGF-2 (Peprotech, London, UK). Cells were passaged weekly by limited trypsinization to generate small clusters of cells and fed on alternate days. Where used, 10 µM Y-27632 was added to cultures for 1 h prior and 24 h post-passage. Conditioned medium from early passage MEF was collected every 24 h for 7 days, filtered and stored.

### Stirred Vessel Bioreactor Culture and Microcarriers

Siliconized 125 cm^3^ Techne glass culture bioreactors (Jencons-Pls, East Grinstead, UK), modified with a HEPA filter, were used in conjunction with a bulb-ended impeller, the speed of which was regulated by a Techne biological stirrer MCS-104S. Three types of microcarriers, Solohill FACT and Solohill Collagen (Sigma, Pode, Dorset, UK; Solohill, Ann Arbor MI), and Cultispher-S (Sigma; Percell Biolytica, Astorp, Sweden) were used in this study, the properties of which are summarized in Supplemental Table S1. Microcarriers were prepared according to the manufacturers' recommendations and the amounts used kept constant throughout the study, namely for each separate culture 50 mg of Cultispher-S and 1 g of Solohill Collagen or Solohill FACT microcarriers were used. Before use microcarriers were conditioned in either KO-DMEM plus KO-SR; GMEM plus serum or MEF-conditioned medium, for a minimum of 30 min. Media volumes in bioreactors were 50 mL (mESC) and 30 mL (hESC). Following seeding optimization, single cell suspensions of mESC or a suspension containing small clusters of hESCs were seeded onto microcarriers at a density of 6 × 10^4^/mL with intermittent stirring of 10 min off, 2 min on at 15 rpm for the first 24 h. After 24 h the impeller speed increased to 45 rpm continuously, the lowest speed that held microcarriers in suspension.

### Sampling

1 mL samples of media, containing suspended microcarriers, were removed for cell counting. To separate unattached cells from those attached to microcarriers, suspensions were filtered through 70 or 100 µm cell strainers (BD Falcon, Oxford, UK). After pelleting, supernatants were retained for bioanalysis, while cell/microcarrier pellets were trypsinized and cells counted in triplicate using Trypan blue exclusion. At the end of each experiment, cells were harvested from microcarriers by trypsinization and set-up in self-renewal assays, or processed for RNA extraction, protein isolation, or immunofluorescence. For analysis of aggregate sizes, samples were photographed and random images selected for measurement. In some cases, particles were separated by passing through 100 µm strainers, prior to image acquisition. This did not affect the size distribution of the aggregates measured, but simplified analyses. In excess of 200 aggregates were measured for each sample and the proportion of aggregates of different sizes determined. Aggregates were defined as clusters of cells with a diameter >49 µm.

### Self-Renewal Assays

The ability of mESC to self-renew was assessed using clonal assays and culture in GMEM supplemented with 10%(v/v) FBS and 1,000 U/mL LIF for 5 days. Alkaline phophatase staining was performed as previously described (Paling et al., 2004).

### Monitoring ESC Metabolic Activity

For each experimental time-point following bioreactor inoculation, the supernatants acquired from sampling were filtered and analytes measured using a Bioprofile 400 Analyser (Nova Biomedical, Waltham, MA). Alternatively, analytes were measured using kits for glucose, lactate, and ammonia (Megazyme, Bray Wicklow, Ireland).

### RNA Isolation and RT-PCR

RNA was isolated and RT-PCR performed as previously described (Storm et al., 2007). The list of primers used in this study are summarized in Supplemental Table S2.

### Protein Extraction and Immunoblotting

Cell extracts were prepared, protein concentrations determined and 20 µg of protein separated by SDS–PAGE and transferred to nitrocellulose as previously described (Welham et al., 1994). Immunoblotting was carried out with the following primary rabbit polyclonal antibodies: 1: 1,000 anti-Nanog (ab 21603; Abcam, Cmbridge, UK); anti-Oct4 (sc9081; Santa Cruz, Santa Cruz, CA) or 1:4,000 anti-Shp2 (sc280; Santa Cruz). An anti-rabbit secondary antibody conjugated to horseradish peroxidase (DAKO) was used for detection and blots were developed using ECL according to the manufacturer's directions (GE Healthcare, Little Chalfont, Bucks, UK). Protein relative quantification was carried out using ImageQuant™ RT-ECL imager and analyzed using ImageQuant™ TL software (GE Healthcare). Blots were stripped and reprobed as previously described (Welham et al., 1994).

### Flow Cytometry and Immunofluorescence

For flow cytometry, TrypLE™ (Invitrogen) dissociated hESC were washed, resuspended in wash buffer (PBS containing 5% (v/v) FCS, 0.1% (w/v) sodium azide) and stained, on ice, with the following antibodies: 1:100 SSEA4 (Abcam, ab16287); 1:100 Tra-1–60 (ab16288; Abcam), or 5:100 phycoerythrin (PE)-conjugated Tra-1–85 (FAB3195P; R&D systems, Abingdon, UK). After washing, samples were incubated for 30 min with secondary FITC-conjugated antibodies (Sigma F1010 & F9259) at 1:100 before being washed again. Flow cytometry was carried out using a FACSCanto (Becton Dickinson, Oxford, UK) and data analyzed using FACSDiva software. Dead cells were excluded from the analysis based on forward and side scatter parameters. For on-microcarrier immunofluorescence cell/microcarriers were fixed with 4% (w/v) paraformaldehyde for 15 min, blocked for 1 h (10% (v/v) Donkey serum, 1% (w/v) BSA in PBS), washed and incubated with 1:20 dilution of PE-conjugated Tra-1–85 (R&D systems, FAB3195P) for 1 h. After washing, DAPI was added for 1 h before samples were mounted on glass slides with fluorescence mounting medium (DAKO). Fluorescence was visualized using Leica DMI 4000B fluorescence microscope.

## Results

### Murine ESCs Cultured on Microcarriers Demonstrate Greater Expansion Than Aggregate Cultures

Based on previous studies (Abranches et al., 2007; Fernandes et al., 2007; Fok and Zandstra, 2005; Kong et al., 1999) and the knowledge that mESCs are routinely cultured on gelatin-coated 2D plates, we selected three different gelatin-based microcarriers (see Table S1) to examine their ability to support the expansion of pluripotent mESCs. Cultispher-S microcarriers are macroporous whereas both Solohill microcarriers consist of cross-linked microporous polystyrene inner cores coated with collagen; Solohill FACT also carry a positive charge. Initial experiments, conducted in static conditions, demonstrated that E14tg2a mESCs attach to all three microcarriers (data not shown). Supplemental movie 1 shows an animation of a Z-stack image of mESCs, stained with DAPI, attached to a Cultispher-S microcarrier. A key aim of our study was to determine the ability of the different microcarriers to support proliferation of ESCs in an agitated suspension 3D system. Stirred vessel bioreactors were chosen since they are (a) suitable for large scale culture (Nienow, 2006); (b) used routinely in industry; and (c) have been used in recent studies with mESCs (Abranches et al., 2007; Fernandes et al., 2007; Fok and Zandstra, 2005) and hESCs (Fernandes et al., 2009; Oh et al., 2009).

**Figure 1:**
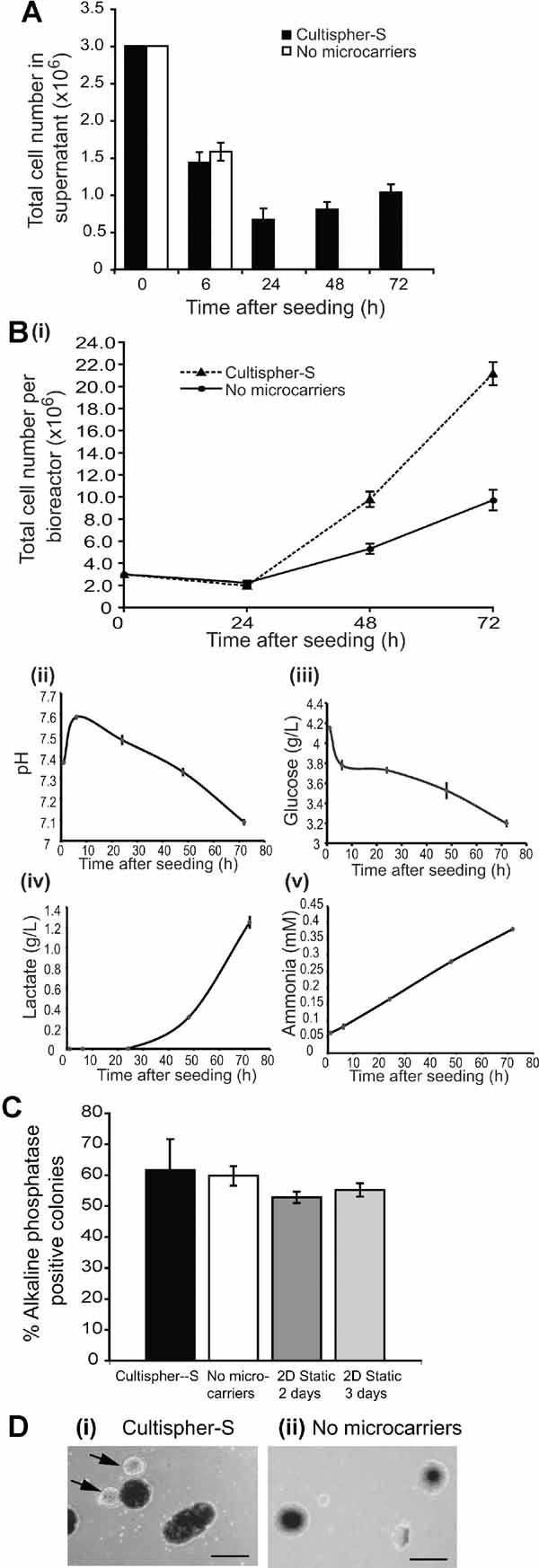
Culture of mESCs on macroporous microcarriers supports their expansion. mESCs were seeded onto Cultispher-S microcarriers at 6 × 10^4^ cells/mL in KO-DMEM plus KO-SR and LIF or directly into bioreactors lacking microcarriers. Impeller speed was continuous at 15 rpm for 2 h and 60 rpm subsequently. A: Average number of live cells remaining in the supernatant are shown (*n* = 3, ±SEM). B: (i) Total number of viable cells attached to microcarriers (dashed line) or in aggregates (solid line) per bioreactor were determined and media was analyzed for (ii) pH, (iii) glucose, (iv) lactate, and (v) ammonia. Mean values are shown (*n* = 3, ±SEM). C: Following 72 h of bioreactor culture, self-renewal of mESCs was assessed and alkaline phosphatase positive colonies scored. Mean values are shown (*n* = 3, ±SEM). “2D Static” refers to E14 mESCs maintained on 2D-tissue culture plates for 2 or 3 days. D: Photographs were taken at 72 h of (i) Cultispher-S-containing cultures and (ii) cultures without microcarriers. Black arrows indicate cell aggregates formed in Cultispher-S cultures, which are smaller and more transparent than the microcarriers themselves and which can attach at a single point to the microcarriers. Scale bar = 200 µm.

For comparision with previous studies, we first examined the culture of mESCs on Cultispher-S microcarriers versus as aggregates. We focused on the first 72 h after seeding so that our procedure reflected as closely as possible standard 2D culture systems for mESCs, where cells are passaged every 48–72 h, while at the same time avoiding the need to passage in these initial investigations. After 24 h culture in bioreactors not containing microcarriers, no cells could be detected in the media supernatant (Fig. 1A), whereas in the presence of Cultispher-S some live cells remained in the media supernatant. Interestingly, when cell expansion was assessed (Fig. 1B(i)), mESCs cultured with Cultispher-S demonstrated a sevenfold increase in cell number compared to an approximately threefold increase in cultures lacking microcarriers, reflecting previous findings (Abranches et al., 2007). Increases in cell number in the Cultispher-S cultures were paralleled by a decline in pH and glucose concentration (Fig. 1B(ii) and (iii)) and increases in lactate and ammonia levels (Fig. 1B(iv) and (v)). As an assessment of pluripotency, the ability of mESCs to self-renew was measured. mESCs were plated at clonal density in the presence of LIF and colonies stained for the presence of alkaline phosphatase; those staining positive were scored as self-renewing. After 3 days of culture there was no significant difference in self-renewal between cells grown on Cultispher-S versus aggregates or controls grown using 2D static conditions (Fig. 1C). Consistent with this observation, >85% of cells were positive for the pluripotency marker Oct4, measured by flow cytometry (data not shown). While these results demonstrate that microcarriers support more rapid mESC proliferation, after 72 h we noticed the presence of variably sized cell aggregates in these bioreactors (Fig. 1D). Possible explanations are either that mESCs fail to attach firmly to the Cultispher-S or that the initial seeding conditions had been sub-optimal, leading to heterogeneity in the culture. We considered such heterogeneity to be undesirable and sought to resolve this issue.

### Cell Seeding Conditions Critically Influence mESC Attachment to Microcarriers and Aggregate Formation

**Figure 2:**
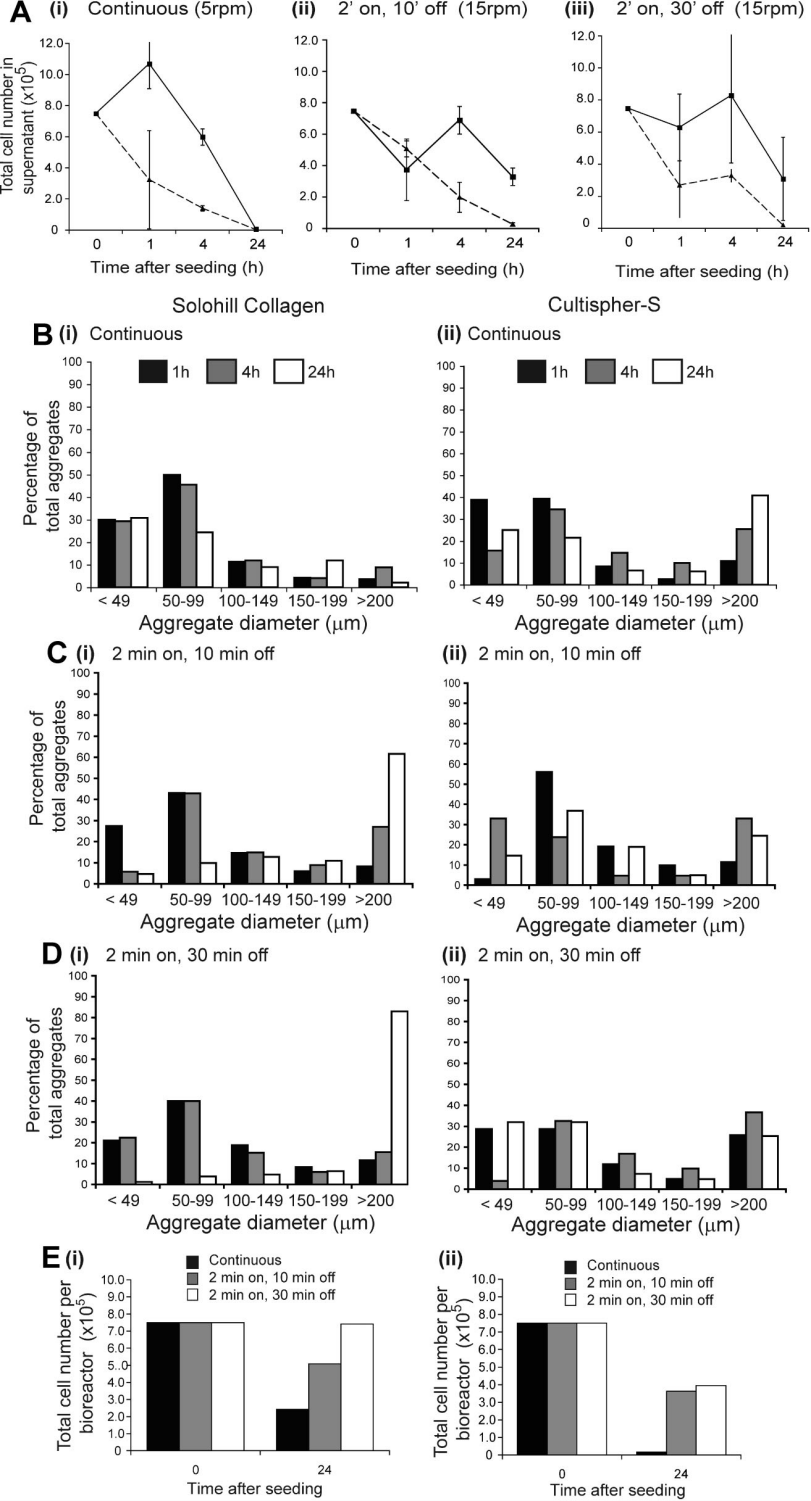
Initial seeding parameters play a critical role in minimizing aggregate formation. A: mESCs were seeded in triplicate onto either Solohill Collagen (solid lines) or Cultispher-S (dashed line) microcarriers at a density of 1.5 × 10^4^ cells/mL in KO-DMEM plus KO-SR and LIF. Seeding was carried out for 24 h with (i) continuous stirring of 5 rpm, (ii) 2 min stirring at 15 rpm followed by 10 min off, or (iii) 2 min stirring at 15 rpm followed by 30 min off. The average number of cells (±SD) remaining in the supernatant are shown. B–D: The diameters of more than 200 cell aggregates were measured and the percentage of aggregates in each of the ranges indicated calculated for each time and condition. B: Continuous stirring of 5 rpm; C: 2 min at 15 rpm, 10 min off, and D: 2 min at 15 rpm, 30 min off. Samples B(i), C(i), and D(i) represent data for Solohill collagen, while B(ii), C(ii), and D(ii) represent data for Cultispher-S. E: The total number of viable cells recoverable from each bioreactor at 24 h was determined (i) Solohill collagen and (ii) Cultispher-S microcarriers.

One of the key aims of our study was to develop robust and homogeneous 3D culturing conditions for ESCs and we reasoned a critical element in achieving this was to optimize initial seeding conditions to promote cell attachment to microcarriers and minimize aggregate formation, parameters which have not been extensively optimized before. Seeding in reduced volumes, pre-treatment of microcarriers with serum and manual agitation were assessed but none improved seeding (data not shown). Next, we compared the effect of seeding mESCs onto either Solohill Collagen or Cultispher-S microcarriers using continuous stirring at 5 rpm, or intermittent stirring of either 10 min off, 2 min on at 15 rpm or 30 min off, 2 min on at 15 rpm, the latter condition having been used in previous studies (Abranches et al., 2007; Fernandes et al., 2007; Fok and Zandstra 2005). We also reduced the starting density of cells from 6 × 10^4^/mL to 1.5 × 10^4^/mL (7.5 × 10^5^ cells in total in a final volume of 50 mL), since a lower cell density would decrease cell–cell interactions, potentially reducing aggregate formation. During the 24 h post-seeding period, we observed a decline in the number of viable mESCs present in the culture supernatant (Fig. 2A). To assess the success of each seeding condition in promoting cell attachment, as opposed to formation of aggregates, we profiled the size of aggregates formed over this timeframe. Samples were harvested and the size of aggregates analyzed. Aggregates, which are spherical and have dense centers fading to the exterior (as shown in Fig. 1D(ii)) were distinguished from microcarriers based on visual appearance; Solohill microcarriers are refractive regularly shaped spheres (Table S1), whereas Cultispher-S have a greater variation in shape and an even optical density. Continuous stirring during seeding minimized the size of aggregates formed in Solohill Collagen, and to a lesser extent Cultispher-S, cultures (Fig. 2B). Intermittent stirring regimes led to a higher frequency of aggregates over the size of 200 µm in the Solohill Collagen cultures (Fig. 2C(i) and D(i)), while this was much less pronounced in the Cultispher-S cultures. Supplemental Table S3 summarizes the mean size of aggregates formed in the different conditions. When the total number of viable cells present in each bioreactor was calculated after 24 h (Fig. 2E) the continuous seeding conditions produced very low cell yields, suggesting that the decrease in cell number observed in the supernatant was due to cell death rather than attachment or aggregate formation. Cell loss, compared to initial numbers seeded, was also observed for the intermittent stirring conditions used. These results highlight the importance of carefully optimizing the initial seeding parameters such that attachment is favored over aggregate formation and/or cell death.

**Figure 3:**
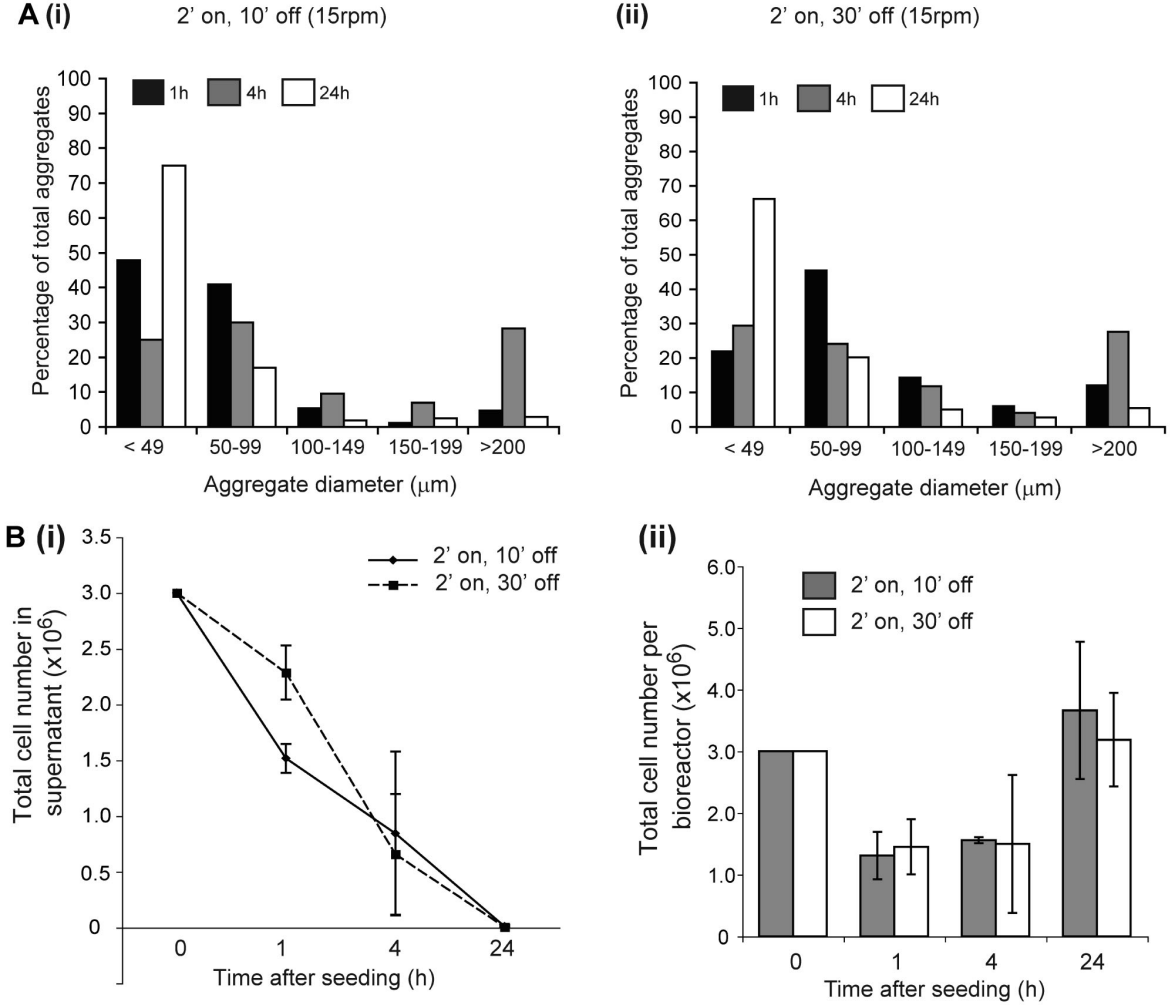
Increased initial seeding density minimizes aggregate formation. A: mESCs were seeded onto Cultispher-S at a density of 6 × 10^4^ cells/mL using either (i) 2 min stirring at 15 rpm followed by 10 min off or (ii) 2 min stirring at 15 rpm followed by 30 min off. Samples were collected and the percentage of aggregates in the ranges indicated calculated for each condition. B: (i) The average number of cells remaining in the supernatant (*n* = 3, ±SD) and (ii) the total number of viable cells recoverable from each bioreactor (mean ± SD, *n* = 3) are shown.

Given the relatively poor cell recovery at 24 h we examined whether increasing the seeding density to 6 × 10^4^/mL would improve this. As shown in Figure 3 (also see Table S3) the majority of aggregates formed in Cultisher-S cultures after 24 h were <49 µm in diameter, irrespective of seeding parameters. Seeding appeared to be more consistent (Fig. 3B(i)) and cell recovery at 24 h was greatly improved compared to seeding at 1.5 × 10^4^/mL (compare Fig. 3B(ii) with Fig. 2E(ii)). These results indicate that a minimum cell density is required during seeding to facilitate effective microcarrier attachment in favor of aggregate formation, an empirical observation that at first seems counter-intuitive. Taking into account the different parameters examined, we decided to seed cells at 6 × 10^4^/mL using intermittent stirring of 2 min on, 10 min off at 15 rpm for 24 h for subsequent investigations.

### Media of Differing Composition Support Expansion of mESCs on Microcarriers

**Figure 4:**
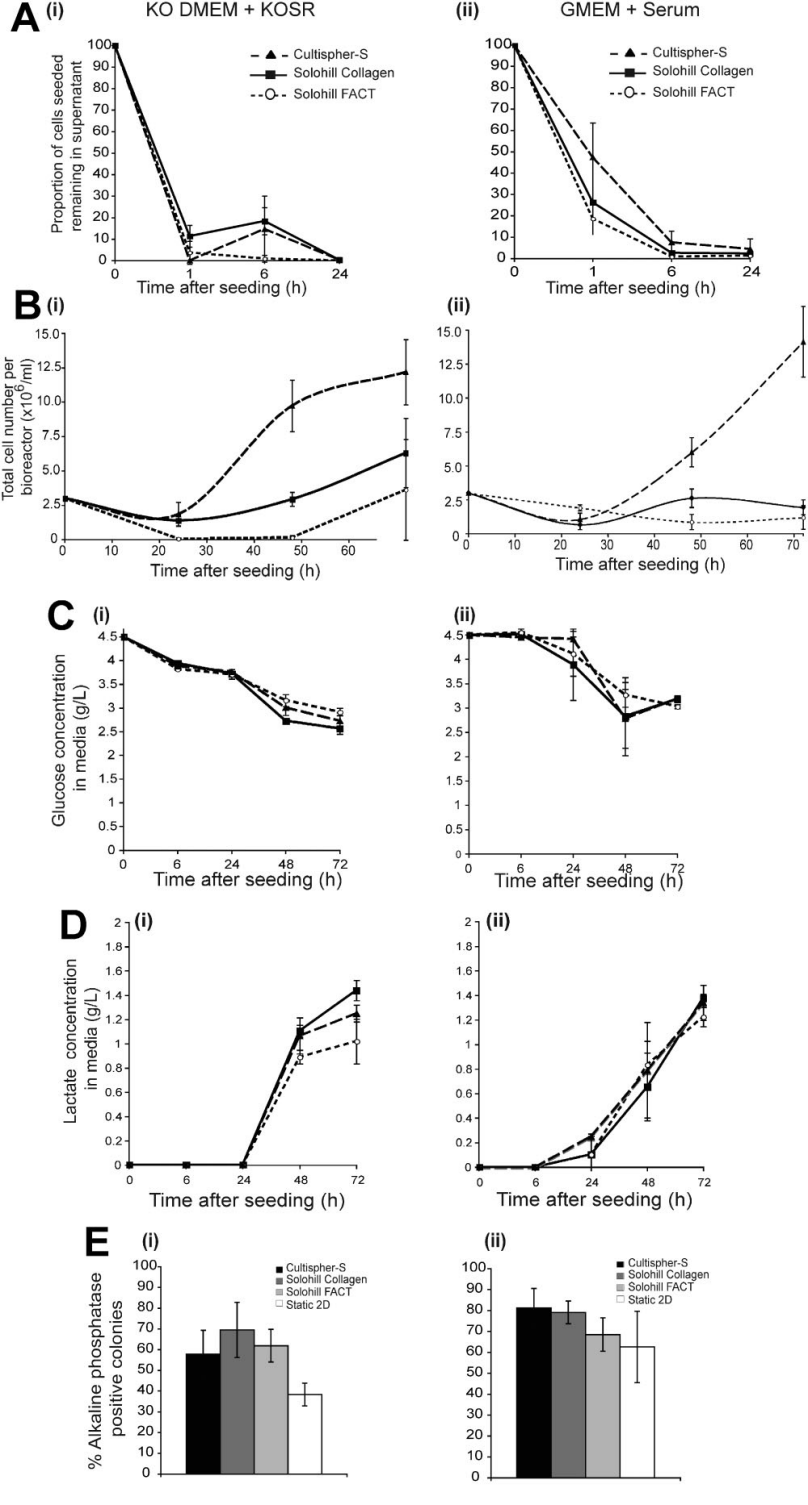
Media of different compositions support expansion of mESCs on microcarriers in bioreactors. mESCs were seeded at 6 × 10^4^ cells/mL in either (i) KO-DMEM plus KO-SR and LIF or (ii) GMEM plus serum (10% v/v) and LIF, using 2 min at 15 rpm followed by 10 min off, onto each of three microcarriers in parallel in duplicate: Cultispher-S (dashed lines, filled triangle), Solohill collagen (solid lines, filled square), or Solohill FACT (dotted lines, open circles). A: The number of cells remaining in the supernatant were determined and the average proportion of cells seeded that remained in the supernatant (±SD) are shown. B: The mean numbers of viable cells attached to microcarriers (*n* = 3, ±SD) are shown for each condition. The mean levels (±SD) of (C) glucose and (D) lactate are shown. E: Self-renewal was assessed after 72 h as described in legend to Figure 1C.

Following seeding optimization, we compared the utility of (i) KO-DMEM plus KO-SR, supplemented with LIF and (ii) GMEM supplemented with serum and LIF to support expansion of pluripotent mESCs on three types of microcarrier. As shown in Figure 4A, seeding of mESCs onto each microcarrier was comparable when the same media was used, although more rapid seeding was noted with KO-DMEM (Fig. 4A(i)). When cell growth was analyzed (Fig. 4B), a lag-phase of approximately 24 h was observed, similar to that reported previously (Fernandes et al., 2007) and maximal cell expansion was observed with Cultispher-S in both media, the total number of viable cells increasing 4-fold and 4.7-fold, respectively. Aggregates present at 72 h had an average diameter of 57.1 µm in KO-DMEM and 24.6 µm in GMEM plus serum, the latter less than the threshold set to define an aggregate. In contrast, cell expansion was low with the Solohill Collagen and poor with the Solohill FACT (Fig. 4B). We also assessed nutrient consumption and metabolite production, allowing us to follow conditions throughout the time-course of cultures. Glucose concentrations fell over time (Fig. 4C), with corresponding increases in lactate production (Fig. 4D), although levels remained below the 1.5 g/L level reported to inhibit growth and induce differentiation of mESCs (Ouyang et al., 2007). Ammonia levels also increased at a steady rate, although remained relatively low (2.2-fold increase to 2.4 mM ± 0.08 (SEM) over 72 h) suggesting they would have little effect on the cultures. Glutamine consumption remained steady, with >50% remaining at 72 h, while CO_2_ saturation and pH remained relatively constant in all bioreactors. These results suggest that while cell proliferation on Solohill microcarriers is less robust than that on Cultispher-S, mESCs present in these cultures are still alive and metabolically active, evidenced by nutrient consumption and metabolite production. Alternatively, it may be that for mESCs metabolite levels are poor indicators of their division and hence growth. When we assessed self-renewal of mESCs cells grown on microcarriers, compared to mESCs grown using standard 2D conditions, an elevation in the proportion of alkaline phosphatase positive colonies was observed (Fig. 4E). These results indicate that short-term growth on microcarriers, irrespective of the microcarrier type, sustains mESC self-renewal.

### Microcarriers Support Long-Term Expansion of Self-Renewing mESCs

**Figure 5:**
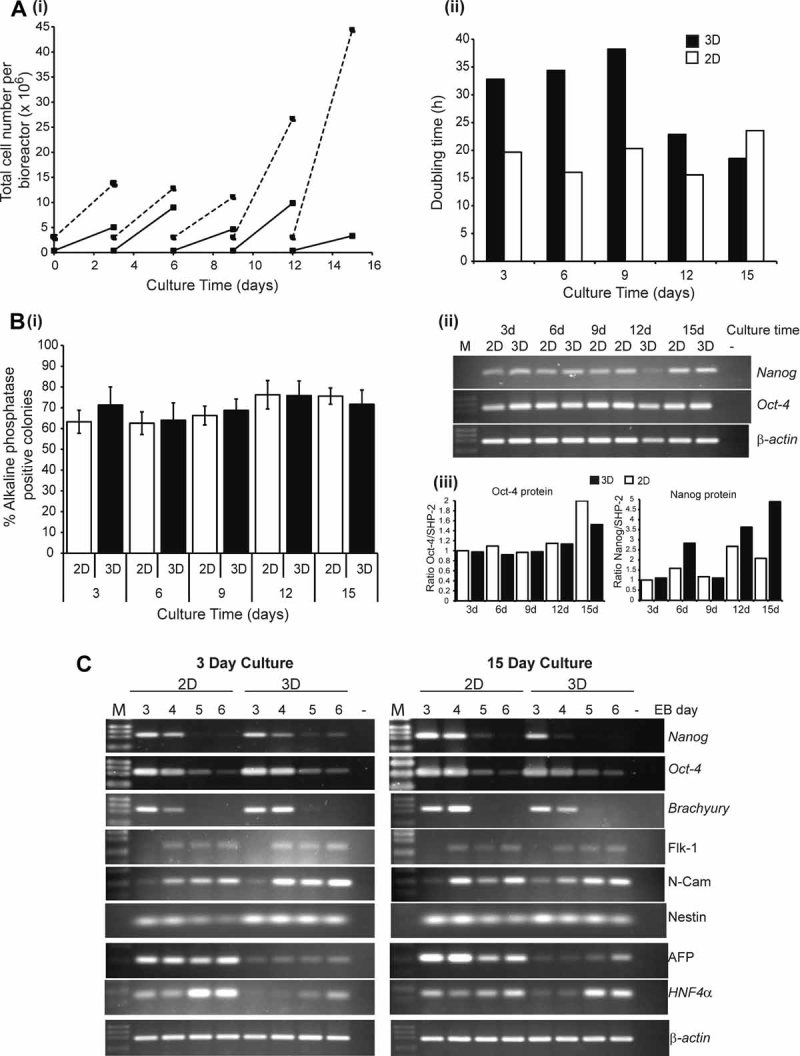
mESCs cultivated long-term on microcarriers retain their undifferentiated state. mESC were seeded onto Cultispher-S microcarriers as described in the legend for Figure 4 (3D samples) or plated onto 10 cm tissue culture plates (2 × 10^5^ total) in the same culture medium (2D samples). Cells were passaged every 3 days and total cell numbers recovered were recorded prior to re-plating using the same conditions described above. A: (i) Cell numbers plated and recovered at each passage for each condition are presented. 2D samples are indicated by the solid line and 3D samples by the dashed line. (ii) Population doubling times for cells in 3D and 2D cultures are shown. B: At each passage cells were trypsined. (i) Self-renewal was assessed and alkaline phosphatase positive colonies scored. Means ± SD from two independent long-term culture experiments for each condition are shown. (ii) RNA or (iii) protein were extracted from cells cultured in 2D or 3D conditions and expression of Oct-4 and Nanog determined by semi-quantitative RT-PCR (ii) or quantitative immunoblotting (iii). C: mESCs cultured in 2D or 3D for 3 or 15 days were plated into methylcellulose to generate embryoid bodies. EBs were harvested, RNA extracted and expression of marker genes analyzed by semi-quantitative PCR. M, molecular markers; −, PCR negative control.

Next, we investigated the ability of mESC pluripotency to be sustained during prolonged culture on microcarriers. mESCs were cultured for a total of 15 days on Cultispher-S and, following trypsinization, passaged onto new microcarriers at 3-day intervals. For comparison, cells were maintained in 2D cultures and passaged at the same times. At each passage, total viable cell numbers were determined as was self-renewal capacity. As shown in Figure 5A(i) cell yields from the first three passages on Cultispher-S were similar to those observed previously (Fig. 4B). As the number of passages increased, so the growth rate of mESCs in the 3D bioreactor approached that observed for cells in 2D culture and population doubling times dropped from 32 to 20 h (Fig. 5A(ii)), the latter comparing favorably to doubling times of mESCs in 2D cultures (18–21 h). We observe no significant differences in self-renewal of mESCs over the time-course (Fig. 5B(i)), consistent with the maintenance of Oct-4 and Nanog RNA (Fig. 5B(ii)) and protein expression (Fig. 5B(iii)), although Nanog protein levels were on the whole elevated in 3D cultures. This latter observation is consistent with our previous results suggesting that Nanog expression may to be related to cell density, resulting either from cell–cell communication or the action of autocrine factors (Storm et al., 2007; Welham et al., 2007). To further examine pluripotency, we plated cells cultured for 3 or 15 days into methylcellulose to facilitate spontaneous differentiation via embryoid bodies. EBs were harvested between 3 and 6 days of development, RNA extracted and analyzed for expression of marker genes, indicative of different lineages. As shown in Figure 5C the temporal patterns of expression of the pluripotency markers Nanog and Oct-4, the mesodermal markers *Brachyury* and Flk-1 and the ectodermal markers, Ncam and Nestin were similar between EBs derived from mESCs grown in 2D or 3D conditions, after both 3 days of culture and 15 days. Interestingly, we consistently observed differences in the pattern of expression of the endodermal marker α-fetoprotein (AFP) between the 2D and 3D-derived EBs. This difference was also apparent, to some extent, for HNF4α expression. Overall, these three lines of evidence demonstrate that pluripotency of mESCs can be effectively sustained following culture of mESCs on microcarriers in agitated suspension in bioreactors.

### Expansion of Human ESCs on Cultispher-S Microcarriers in Agitated Suspension Bioreactor Cultures

**Figure 6:**
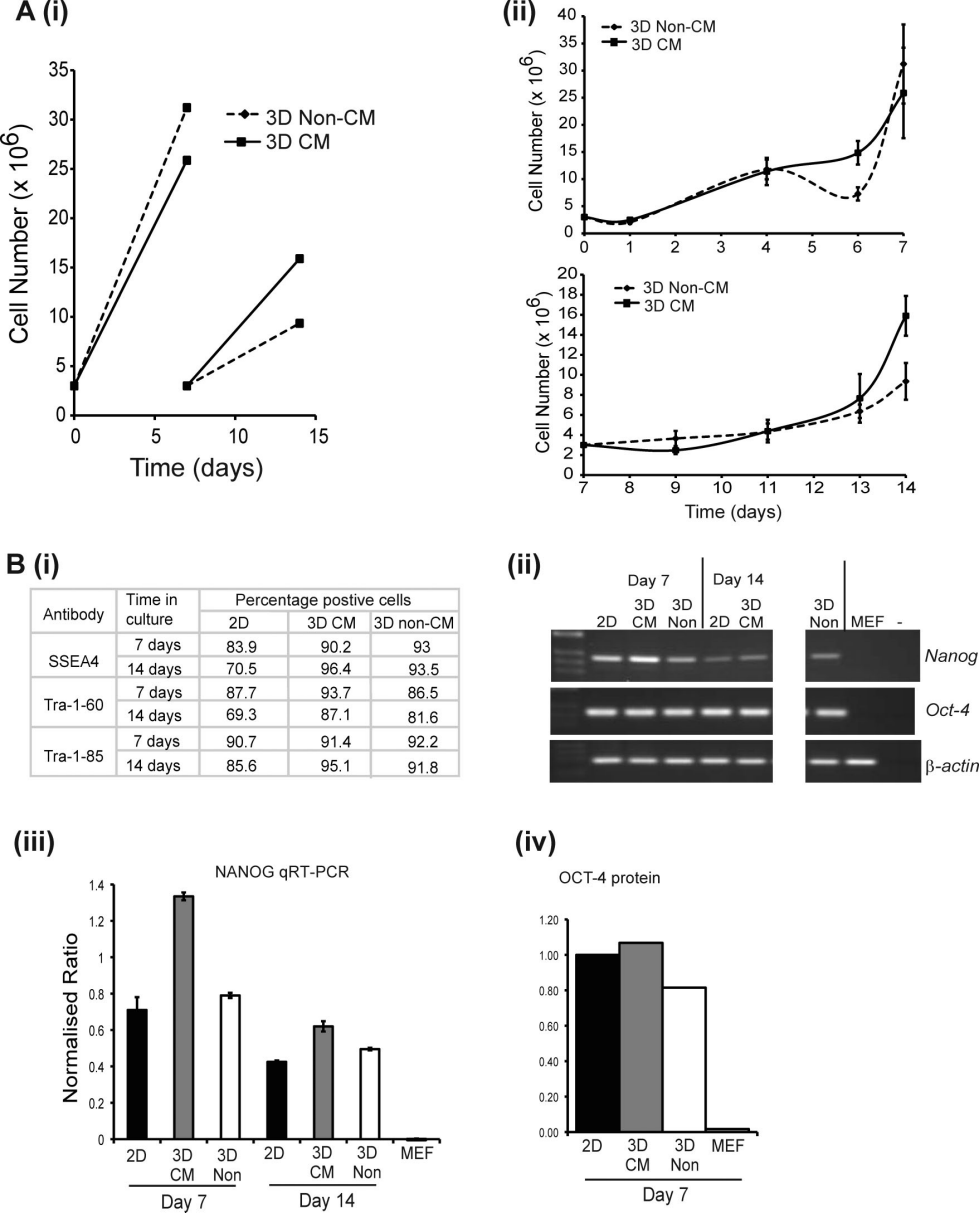
Cultispher-S microcarriers support expansion of undifferentiated SHEF3 hESCs. 3 × 10^6^ SHEF3 hESCs were seeded onto Cultispher-S microcarriers in KO-DMEM supplemented with 20% (v/v) KO-SR and 4 ng/mL FGF-2 in the presence (3D CM) or absence (3D Non-CM) of MEF conditioned medium (CM). Cells were cultured for 7 days, passaged and then cultured for a further 7 days. A: (i) Cell numbers plated and recovered at each passage for each condition are presented. (ii) The mean number of viable cells attached to microcarriers (±SD) are shown for each condition for the culture periods d0–d7 (upper panel) and d7–d14 (lower panel). B: Assessment of markers of pluripotency. (i) Expression of SSEA4, Tra-1–60 and Tra-1–85, were determined by flow cytometry. 2D indicates hESCs controls maintained in 2D culture on MEF plus FGF-2. RNA and protein were extracted from hESCs cultured in 2D or 3D conditions for the times indicated and expression of OCT-4 and NANOG determined by (ii) semi-quantitative RT-PCR; (iii) quantitative RT-PCR normalized to β-actin (±SD) or (iv) immunoblotting, normalized to SHP-2.

Based on our success in expanding pluripotent mESCs on microcarriers in bioreactors, we wanted to test whether we could apply similar parameters to expansion of hESCs. Survival of hESCs when passaged as single cells is poor but we wanted to avoid any pre-selection, since it can contribute to chromosomal changes associated with culture adaptation and improved growth (Draper et al., 2004). Therefore, we initially assessed the ability of hESCs to attach to and expand on three different microcarriers in conjunction with different dissociation regimes. In addition, based on reports that inhibition of Rho kinase improves hESC survival (Watanabe, 2007), we included the ROCK inhibitor, Y-27632, at 10 µM during cell passage. Initial results, shown in Supplemental Figure S1, demonstrated preferential attachment and expansion of Accutase-dissociated hESC on Cultispher-S. Next, SHEF3 hESCs were seeded onto Cultispher-S microcarriers (no Matrigel coating was employed) using the seeding parameters optimized for mESCs and cultured in stirred bioreactors in the presence of either FGF-2 and MEF conditioned medium (3D CM samples) or in the presence of FGF-2 only (3D Non-CM samples). For comparison a portion of hESCs were maintained in 2D culture in the presence of FGF-2 on a MEF feeder layer. Media was partially exchanged at 2-day intervals and cells were passaged at day 7. Pooled cell populations from each culture condition were reseeded onto fresh Cultispher-S and cultured for a further 7 days (14 days in total). hESC expansion was evident in the presence and absence of MEF conditioned medium (see Fig. 6A(ii) and (ii)), although we noticed an apparent decline in expansion during the second 7 days, correlating to an apparent increase in lag-phase (compare upper and lower panels in Fig. 6A(ii)). This may have been due to a reduction in the size of hESC clusters generated following accutase treatment at this step, which would have led to reduced cell viability and so the total number of cells available for attachment. To assess pluripotency of the expanded hESCs, we analyzed cell surface expression of the markers SSEA4 and Tra-1–60, as well as the pan-human marker Tra-1–85, an indicator of the proportion of hESCs present (Fig. 6B(i) and Supplemental Fig. S2). RNA and protein expression of the pluripotency markers Nanog and Oct-4 were also assessed (Fig. 6B(ii–iv)). 3D hESC cultures in the presence of MEF CM maintained expression of all pluripotency markers examined, which in some cases were higher than comparative 2D controls (at day 7 Nanog was 1.85-fold higher in 3D CM samples compared to 2D control and 1.5-fold higher at day 14). These data provide some of the first evidence that previously unselected hESCs, that retain expression of markers of pluripotency, can be expanded and serially passaged on microcarriers using stirred bioreactors.

**Figure 7:**
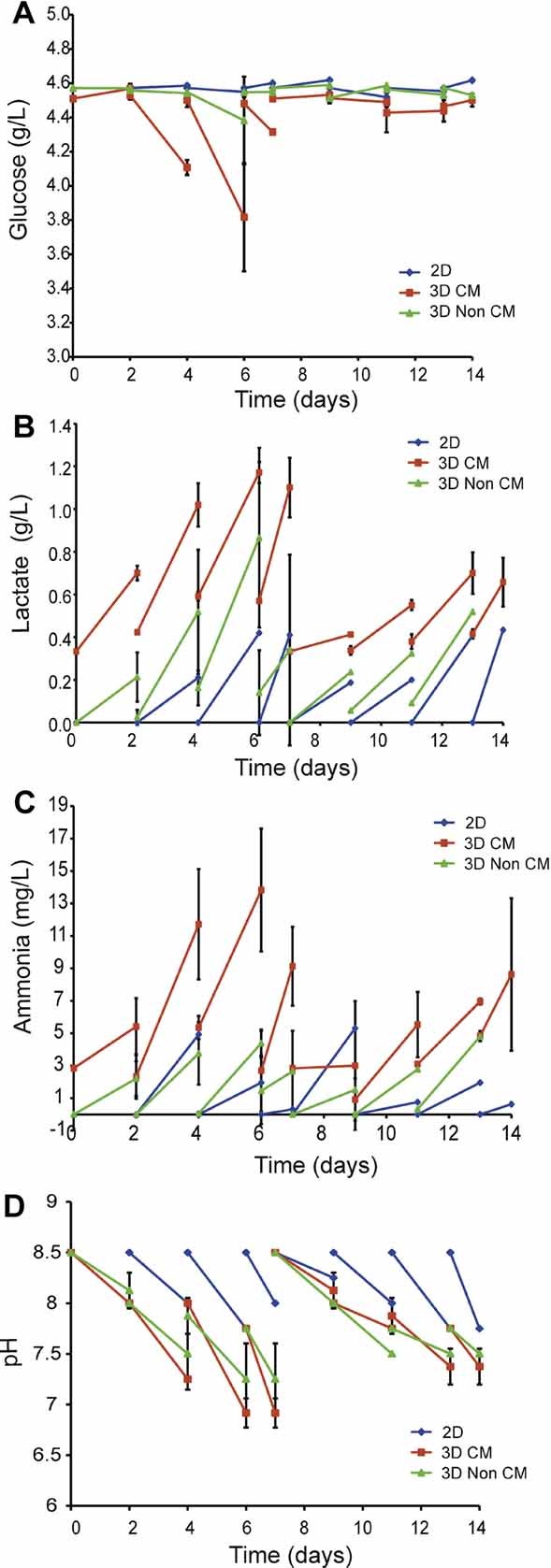
Nutrient and metabolite profiles of SHEF3 hESC-Cultispher-S bioreactor cultures. SHEF3 hESCs were seeded onto Cultispher-S. Media samples were harvested and analyzed for the concentration of: (A) glucose; (B) lactate, and (C) ammonia. D: The pH of media samples. Triplicate measurements were taken from duplicate samples for MEF CM and Non-CM conditions during the first 7-day culture period, averages ± SD are shown. 2D samples are a single replicate. [Color figure can be viewed in the online issue, which is available at http://www.interscience.wiley.com.]

To gain further insight into the characteristics of culturing hESCs in agitated suspension in bioreactors, we analyzed nutrient and metabolite levels. hESCs cultured in MEF CM demonstrated the biggest consumption of glucose during the first 7 day culture period (Fig. 7A), mirrored by increased production of lactate (Fig. 7B) and ammonia (Fig. 7C). The elevated basal level of lactate and ammonia observed is due to the use of conditioned medium, which already contains increased levels of these cell metabolites. Lactate and ammonia production closely paralleled cell growth profiles (see Fig. 6A(ii)), indicating that these are good indicators of hESC behavior in bioreactor cultures, whereas glucose consumption appeared to be less reliable. Possible explanations are either that hESC metabolism differs from somatic cells or, the extended lag-phase observed has resulted in low glucose consumption. Corresponding decreases in pH were observed over the timeframe of experiments, although the pH (Fig. 7D) did not drop considerably below 7.

## Discussion

Scale-up of ESC culture is necessary in order to support future developments in the application of hESCs and their differentiated derivatives in regenerative medicine and drug discovery. To address this, we sought to develop a robust 3D culture platform for the expansion of pluripotent ESCs. Using mESCs as our initial model, we demonstrate the ability of three different microcarriers to support their expansion. Our studies clearly highlight the importance of the initial seeding parameters in promoting cell-microcarrier attachment and minimizing the size of aggregates formed, factors that had not been considered in depth before. Using our optimized conditions, pluripotent mESCs could be serially passaged over a 15-day period. Importantly, particularly from the translational and clinical perspective, the conditions optimized using mESCs could be directly transferred to hESCs previously cultured on a feeder layer, without the need for weaning or adaptation. Using these conditions, we demonstrate the successful direct seeding of hESCs into Cultispher-S microcarriers in stirred bioreactors. hESCs cultured in this system could be passaged onto fresh microcarriers and expanded further while maintaining expression of pluripotency markers. Our study highlights the importance of careful optimization of initial seeding parameters, the utility of mESCs as a valuable model for platform development in this area and the ability to directly seed hESCs onto microcarriers in a stirred bioreactor without prior selection, effectively simplifying bioprocess requirements.

The advantage of 3D bioreactor cultures is that manual processing can be minimized and culture volumes readily expanded. One concern that arose early in our studies was the appearance of aggregates, leading to culture heterogeneity. While it has been reported that even after extensive suspension culture as aggregates mESCs maintain expression of pluripotency markers (Cormier et al., 2006; Fok and Zandstra 2005; zur Nieden et al., 2007), aggregate formation is most often associated with induction of differentiation via embryoid bodies (Keller, 2005), so we considered it undesirable to culture ESCs in this way for scale-up purposes. Thus, we sought to minimize aggregate formation and found that careful optimization of the initial seeding conditions, including cell number and agitation, was necessary to promote cell-microcarrier attachment and minimize the size of aggregates formed. This attention to detail in the developmental stages of our study led to successful translation of conditions from mESCs directly to hESC cultures. Our study represents one of the first reports, along with (Fernandes et al., 2009; Oh et al., 2009) that demonstrates direct seeding of hESCs onto microcarriers in an agitated suspension bioreactor system.

The culture of mESCs on microcarriers has been previously reported (Abranches et al., 2007; Fernandes et al., 2007; Fok and Zandstra 2005) and during the first 72 h of culture, the 7-fold expansion we observe is similar to the ∼5-fold and 10-fold expansion observed by Abranches et al., 2007 and Fernandes et al., 2007, respectively. Our studies demonstrate that initial population doubling times of mESCs grown on microcarriers are increased compared to static 2D cultures. However, after three serial passages, doubling times decreased and approached those of cells cultured in 2D, suggesting similar rates of growth can be achieved. We think it highly unlikely that abnormal cells would have been selected for within this 15-day period and our favored explanation, supported by the similarities in levels of self-renewal (Fig. 5B(i)) and differentiation (Fig. 5C), is that the mESCs have adapted to the 3D culture system within this time-frame. In view of the fact that the decline in Nanog and Oct-4 and temporal patterns of expression of mesodermal and ectodermal markers were very similar in EBs generated from 2D- or 3D-cultured mESCs, we were surprised that the pattern of expression of endodermal markers differed. However, these observations may be explained by the fact that 2D culture favors the spontaneous differentiation of mESCs to primitive endoderm that expresses AFP and HNF4α. Thus, expression of these markers is retained during the early stages of EB differentiation from 2D-cultured mESCs. In contrast, culture on Cultispher-S does not lead to the same level of spontaneous differentiation to primitive endoderm, so levels of AFP and HNF4α are lower in days 3 and 4 EBs and rise as differentiation of definitive endoderm progresses from day 5 onwards.

In the past year, a handful of papers have appeared reporting proof-of-concept studies demonstrating the ability of hESCs to attach to and expand on microcarriers. The studies of Phillips et al. (2008) and Nie et al. (2009) focussed on using microcarriers to culture hESCs in a static environment. Phillips et al., found that only ESI-107 hESCs weaned to single cell passaging attached effectively to Hillex II microcarriers, whereas unweaned single cells did not attach at all. In contrast, using SHEF3 hESCs and including the ROCK inhibitor Y-27632 during passage, we were able to avoid any requirement for weaning. This is important because prolonged weaning/selection for hESCs that can tolerate single cell passaging could result in epigenetic or genetic changes, known to arise as a result of culture conditions (Draper et al., 2004) and undesirable for potential clinical applications. Because, we only cultured our hESCs for 2 weeks on Cultishper-S we did not assess their karyotype, but clearly it would be important to do so in future long-term culture studies. Cytodex-3 (Fernandes et al., 2009) and Matrigel and MEF-coated cellulose microcarriers (Oh et al., 2009) have been combined with hESCs and cultured in stirred bioreactors. Fernandes et al., reported a lag-phase of around 200 h with an eventual population doubling time of 75 h during the exponential phase, leading the authors to suggest further “adaptation” of hESCs to feeder-free culture may be required. Based on our observations, an alternative explanation for their results is that initial cell seeding onto the microcarriers was sub-optimal, resulting in low viability. In our system, we estimate that the population doubling time of hESCs on Cultispher-S to be 40.7 h (SE ± 13.8), while Oh et al. (2009) report a doubling time of 21 h. This variation may be due to a number of factors, including the different hESC lines studied, type of microcarriers used, any pre-coating applied, for example, Matrigel, which we did not use, the type of culture media, seeding parameter and densities. Clearly, further optimization and longer-term investigations are required to build on these initial studies and develop the systems more fully. In addition, it will be important to assess pluripotency more fully, not just examining expression of key markers, as we have done, but also by using models of differentiation, for example, embryoid body-based methods and teratoma formation.

Another novel aspect of our study is that we have shown, in principle, that it is possible to passage unweaned hESCs from microcarriers to fresh microcarriers and maintain expression of pluripotency markers. Based on the report that >98% of hESCs cultured for 11 successive passages on microcarriers in static environments retain OCT-4 staining (Nie et al., 2009), similar maintenance of pluripotency may also be achievable following extended passage of hESCs on microcarriers in bioreactors. We have further shown that there is a wealth of nutrient and metabolic data that can be collected during ESC culture. At the simplest level this gives us information on the metabolic state of the cells with time and as we move toward larger-scale cultures in bioprocessing environments, such data will be invaluable for culture design, control, and optimization.

In summary, our study is among the first reporting proof-of-principle data showing that hESC can be expanded on microcarriers in bioreactor cultures. Furthermore, it validates the use of mESCs as a model for this system and provides information pertinent for future developments in the field. With the likely numbers of hESCs required for applications running into the billions (Ouyang and Yang, 2008) only 3D systems offer effective solutions to scale-up hESC culture.
