# Supplementary material for: Three-dimensional culture systems for the expansion of pluripotent embryonic stem cells
Source: Biotechnol Bioeng. 2010 Nov 1;107(4):683–95. doi: 10.1002/bit.22850 (PMC3580883; doi:10.1002/bit.22850)

**Storm et al., Supplemental Table S1. Summary of the microcarriers used in this study**

| ***Microcarrier*** | ***Maker*** | ***Type*** | ***Matrix*** | ***Coating*** | ***Charge*** | ***Density (g/ml)*** | ***Size (µm)*** | ***Area (cm^2^/g)*** | ***Load (g/L)*** |
| --- | --- | --- | --- | --- | --- | --- | --- | --- | --- |
| Collagen | Solohill | Solid | Polystyrene, cross-linked | Type I porcine collagen | None | 1.03 | 150-210 | 325 | 20 |
| FACT | Solohill | Solid | Polystyrene,  cross-linked | Type I porcine collagen | + | 1.03 | 150-210 | 325 | 20 |
| Cultispher-S | Percell Biolytica | Porous | Gelatin |  | None | 1.04 | 170-270 | Not reported | 1 |

Solohill Collagen Solohill FACT Cultipher-S


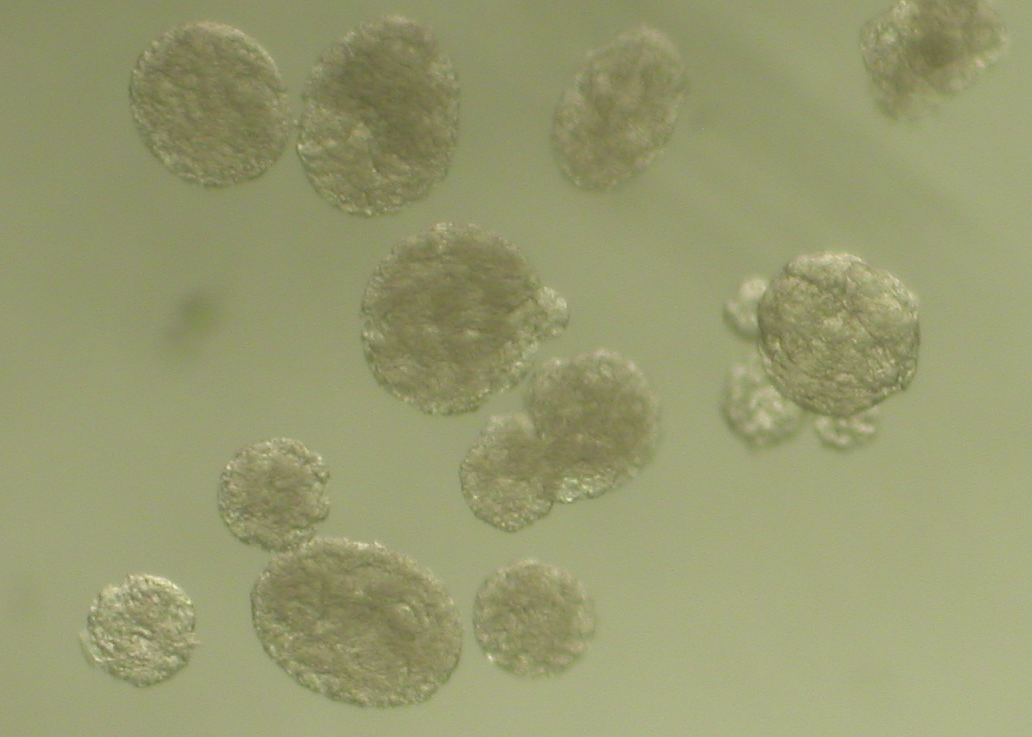

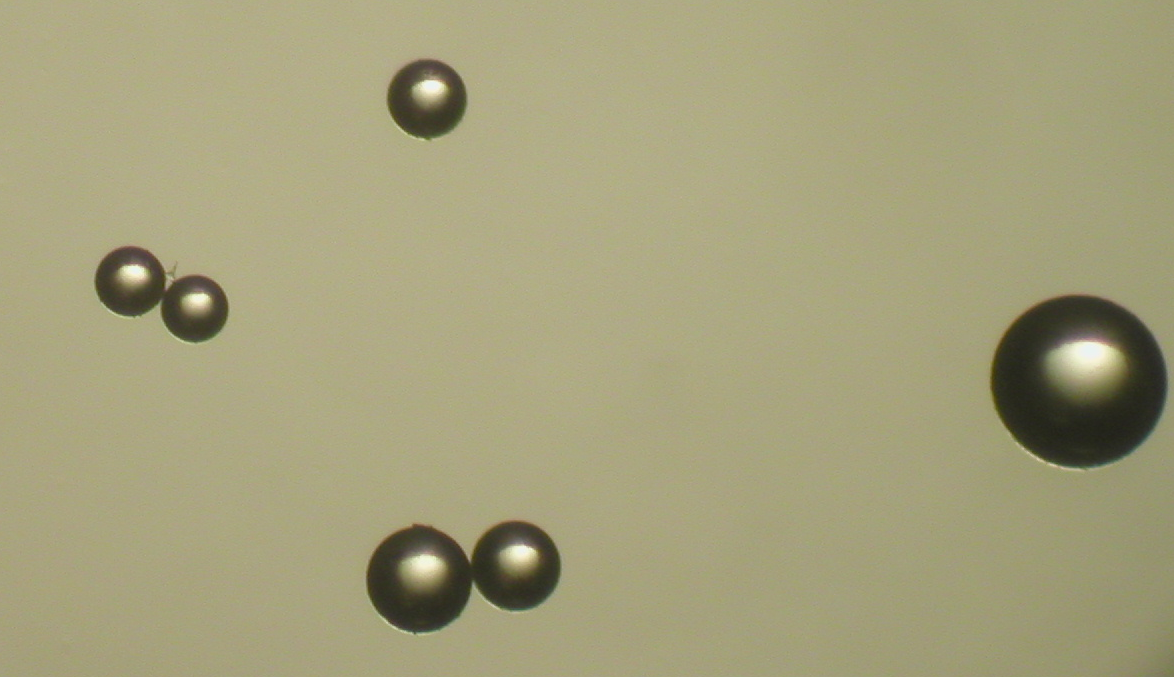

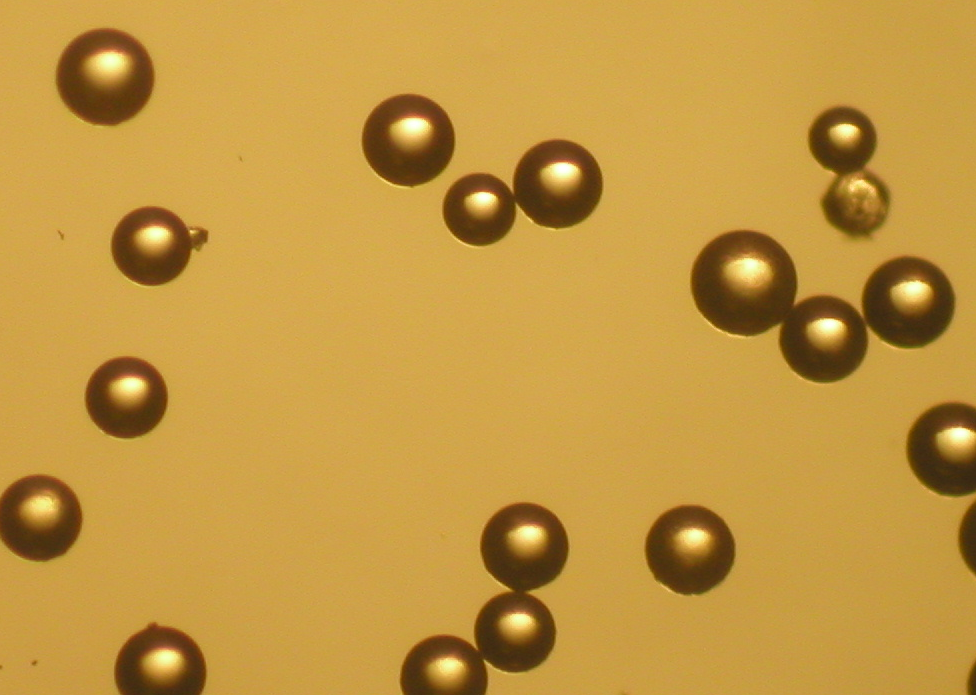


**Storm et al., Supplemental Table S2. The primers used in this study.**

| **Organism** | **Gene** | **Primer sequence (5'-3')** | **Annealing Temp (°C)** | **Cycles** |
| --- | --- | --- | --- | --- |
| Mouse | Nanog | F: CTCTTCAAGGCAGCCCTGAT | 60 | 28 |
|  |  | R: CCATTGCTAGTCTTCAACCAC |  |  |
|  | Oct4 | S: GCGTTCTCTTTGGAAAGGTGTTC | 58 | 25 |
|  |  | AS: CTCGAACCACATCCTTCTCT |  |  |
|  | Brachyury | S: CATGTACTCTTTCTTGCTGG | 58 | 30 |
|  |  | AS: GGTCTCGGGAAAGCAGTGGC |  |  |
|  | Flk1 | S: CACCTGGCACTCTCCACCTTC | 60 | 30 |
|  |  | AS: GATTTCATCCCACTACCGAAAG |  |  |
|  | AFP | S: TCCAGACAAAGAGAGCATCC | 59 | 30 |
|  |  | AS: ACTTTCCTTGGCAACACTCC |  |  |
|  | HNF4α | F: ACAGGAGAGGGTCAGAAGCA | 58 | 30 |
|  |  | R: GATGTTTGCACAACCACAGG |  |  |
|  | Ncam1 | S: CTCGAAGTTCAATATGCTCC | 59 | 30 |
|  |  | AS: CTGAATCAGGGGTCACCTCC |  |  |
|  | Nestin | S: AGGAACCAAAAGAGGCAGGT | 60 | 35 |
|  |  | AS: TTGGGACCAGGGACTGTTAG |  |  |
| Mouse & Human | β-actin | F: TAGGCACCAGGGTGTGATGG | 60 | 25 |
|  |  | R: CATGGCTGGGGTGTTGAAGG |  |  |
| Human | hNANOG | S: CCTGATTCTTCCACCAGTCCC | 65 | 28 |
|  |  | AS: GTCGGGTTCACCAGGCATCCC |  |  |
|  | hOCT4 | S: TGAGGGTGAAGCAGGAGTCGG | 58 | 28 |
|  |  | AS: AAGATTTTCATTGTTGTCAGC |  |  |

**Storm et al., Supplemental Table S3.** The mean diameters, in μm, of the aggregates formed in each of the initial seeding conditions onto Solohill Collagen and Cultispher-S microcarriers, at different cell densities, are summarized.

| **Seeding conditions** | **Solohill Collagen**  **(1.5 x 10^4^/ml)** | **Cultispher-S**  **(1.5 x 10^4^/ml)** | **Cultispher-S**  **(6 x 10^4^/ml)** |
| --- | --- | --- | --- |
| **Continuous** | 133 | 256 | N.D. |
| **2’ on 10’ off** | 299 | 170 | 59.6 |
| **2’ on 30’ off** | 645 | 193 | 74 |


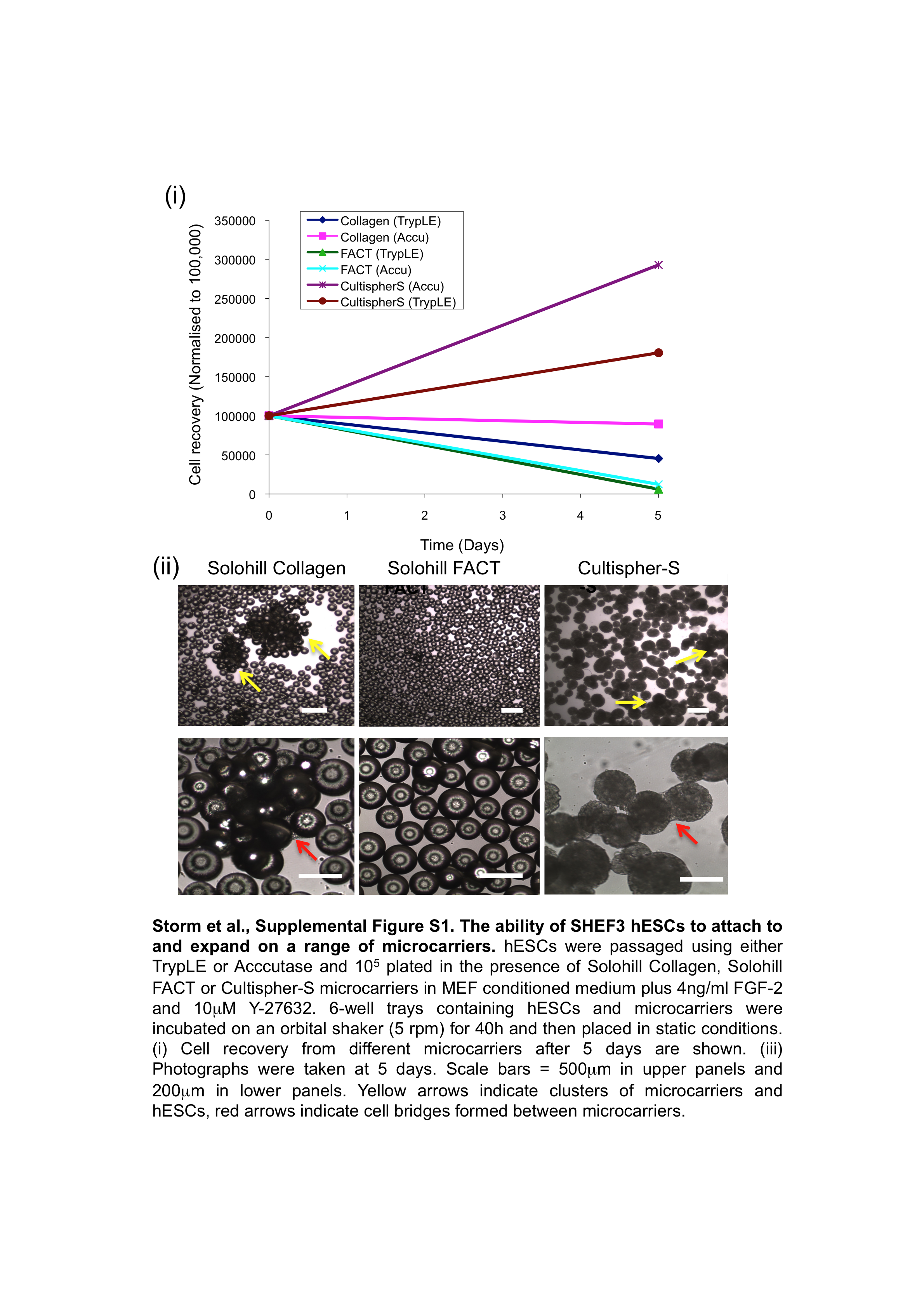


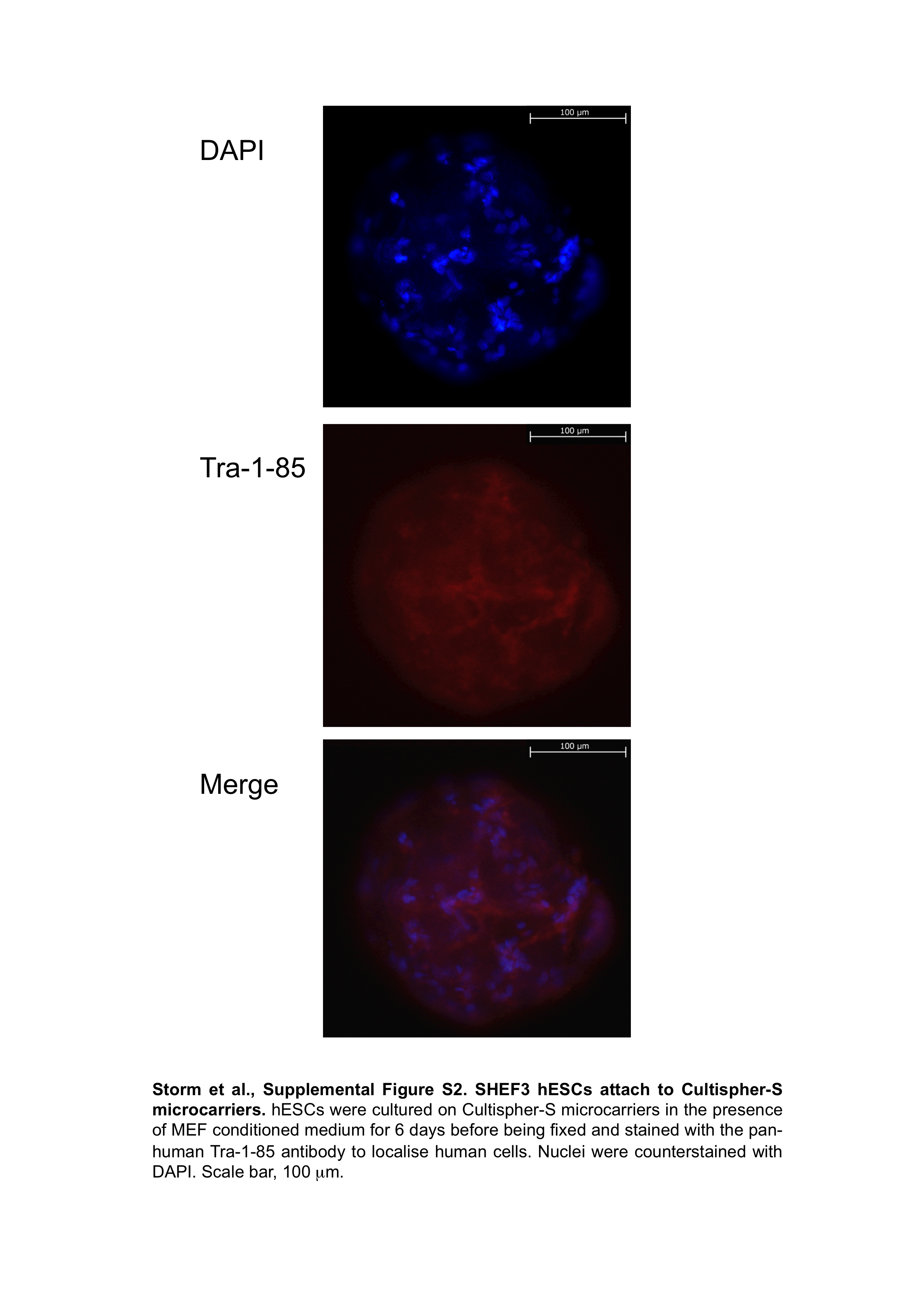

Supplement: Supplementary file 1 [file bit0107-0683-sd1.doc]
